# Climate change and vector-borne disease in North America and Europe

**DOI:** 10.25646/10393

**Published:** 2022-08-31

**Authors:** Lyle R. Petersen, Karen Holcomb, Charles B. Beard

**Affiliations:** 1 U.S. Centers for Disease Control and Prevention, Division of Vector-Borne Diseases Fort Collins, USA; 2 U.S. National Oceanic and Atmospheric Administration, Global Systems Laboratory, Boulder, USA

Global surface temperature, which has already increased approximately 1°C relative to that in 1850 to 1900, may increase another 2 to 4°C by the next century. Precipitation in the United States (US), Canada and Europe will mostly increase, while the southwestern US and southern Europe will become drier. These changes are expected to alter the ecology and transmission dynamics of West Nile virus (WNV), the leading mosquito-borne infection in these regions [1], and Lyme disease and tickborne encephalitis, the respective leading tickborne diseases.

Temperature and rainfall differentially influence many factors related to WNV transmission. For example, increased temperature can increase mosquito development rate and vector competence but can also increase mosquito mortality and decrease the quantity of mosquito breeding sites. Rainfall can increase mosquito breeding sites, but excessive rain eliminates them [2]. Region-wide analysis suggests WNV incidence is highest in areas of the US and Europe with average summer temperatures of 23 to 25°C and that within these areas, heat waves promote outbreaks. In 2018, the largest outbreak in Europe occurred during the hottest summer on record and recent temperature increases have accompanied WNV’s northward spread into Germany and the Netherlands. However, the largest local WNV outbreak occurred in 2021 in Phoenix, Arizona, an area typically experiencing summer temperatures over 30°C, during a period of exceptional summer rainfall. Overall, evidence suggests that increased temperature and rainfall promote WNV incidence in cooler and drier areas, and increased rainfall decreases incidence in wetter areas.

*Ixodes* spp. ticks, the vectors of Lyme disease and tickborne encephalitis, have multi-year life cycles influenced by temperature and precipitation. Warmer temperatures increase tick reproductive capacity, lengthen seasonality, and promote questing, while increased humidity increases survival. Conversely, excessive temperatures and low humidity may increase tick mortality. Climate-related increased temperatures in northern US and Europe have correlated with dramatic northern spread of *Ixodes* spp. ticks and accompanying increase in disease incidence. In Europe, increased temperatures have led to tick migration to higher altitudes. However, in the US, *Ix. scapularis* has also migrated southward, indicating the importance of other ecological factors such as increasing deer populations [3].

In summary, accumulated data indicate that climate change has already impacted vector-borne disease incidence and distribution in the US and Europe and will continue to do so. However, the uncertainty of climate models combined with the differential effects of temperature and rainfall on vector transmission hamper our ability to model future WNV climate-related scenarios. Further investments in environmental data collection and disease/climate modelling efforts applicable to future climate scenarios should be a priority.

*Disclaimer*: The findings and conclusions in the presentation are those of the authors and do not necessarily represent the views of the Centers for Disease Control and Prevention.
